# Comparative study on the efficacy and safety of different left atrial appendage occluders in one-stop atrial fibrillation procedures

**DOI:** 10.3389/fcvm.2026.1742519

**Published:** 2026-05-26

**Authors:** Haoqing Ren, Hengli Lai

**Affiliations:** 1 Jiangxi Medical College, Nanchang University, Nanchang, China; 2 Department of Cardiology, Jiangxi Provincial People's Hospital, The First Affiliated Hospital of Nanchang Medical College, Nanchang, China

**Keywords:** atrial fibrillation, device-related thrombosis, left atrial appendage occlusion, occluder, one-stop procedure

## Abstract

**Objective:**

To compare the sealing efficacy and safety of three left atrial appendage (LAA) occluders—WATCHMAN, LAmbre, and LACBES—in a one-stop procedure combining catheter ablation (CA) and LAA occlusion (LAAO) for atrial fibrillation (AF).

**Methods:**

We retrospectively analyzed 231 AF patients undergoing a one-stop procedure, grouped by implanted occluder (WATCHMAN *n* = 110, LAmbre *n* = 72, LACBES *n* = 49). The 6-month peri-device leak (PDL) was compared across devices, and Firth-corrected logistic regression and Cox proportional hazards models were applied for PDL and stroke analyses, respectively, given low event counts.

**Results:**

Baseline characteristics were comparable across the three groups (*P* > 0.05). PDL rates were 8.2% (9/110), 6.9% (5/72), and 4.1% (2/49) for the WATCHMAN, LAmbre, and LACBES groups, respectively, with no statistically significant intergroup differences (*P* > 0.05). Multivariate analysis using Firth correction confirmed that device type was not an independent predictor of PDL, after adjusting for clinical risk profiles. However, a higher CHA_2_DS_2_-VASc score was an independent risk factor, whereas older age was a protective factor. No statistically significant differences in DRT or stroke incidence were observed among the devices during the study's follow-up period and with its limited sample size. However, the statistical power for these rare safety endpoints was low, necessitating larger studies to confirm long-term safety comparisons.

**Conclusion:**

In the context of the one-stop AF procedure, the WATCHMAN, LAmbre, and LACBES occluders demonstrated comparable effectiveness in achieving LAA closure. A high CHA_2_DS_2_-VASc score and younger age may be risk factors for PDL, warranting closer monitoring in these patient subgroups.

## 1 Introduction

Atrial fibrillation (AF) is one of the most common clinical arrhythmias, with its most severe complication being stroke. Percutaneous left atrial appendage occlusion (LAAO) has become a crucial preventive strategy for AF patients at high stroke risk who have contraindications to long-term oral anticoagulation or a high bleeding risk (1–4). In recent years, the “one-stop” procedure—the concomitant performance of catheter ablation (CA) and LAAO—has emerged as an integrated therapeutic option for AF patients requiring both symptom control and stroke prevention (5–7). Various LAA occluders based on different design concepts are commercially available, such as the plug-based WATCHMAN device, the clamp- and disc-based LAmbre device, and the domestically developed LAmbre device in China, the LACBES (8–12). Existing research has largely treated CA and LAAO as separate procedures, with device comparisons predominantly based on standalone LAAO studies (8, 13–16). However, the one-stop procedure is more than the simple sum of its parts. Successful ablation may create a unique “stable atrial” environment for subsequent occluder implantation by reducing atrial load and improving hemodynamics and electrophysiological substrate. This fundamental shift in the implantation milieu could potentially reshape device performance and compatibility requirements—for instance, by attenuating mechanical stress on the occluder caused by vigorous atrial contractions, thereby narrowing the inherent differences in peri-device leak (PDL) and thrombotic risk among different device designs. Nevertheless, this pivotal hypothesis remains inadequately validated. Furthermore, while existing evidence focuses predominantly on devices of European and American origin (e.g., WATCHMAN, Amulet), there is a lack of head-to-head comparison data for domestically produced LAA occluders like LACBES, despite their innovative designs and growing clinical application, particularly within the complex context of the one-stop procedure.

Therefore, this study aims to move beyond simple device comparison by utilizing advanced statistical methods to investigate, for the first time, whether the efficacy and safety differences among various device designs (WATCHMAN, LAmbre, LACBES) are attenuated within the synergistic therapeutic framework of the one-stop procedure, thereby providing the first evidence-based rationale for “procedure mode-optimized device selection”.

## 2 Materials and methods

This retrospective observational study was reported in accordance with the Strengthening the Reporting of Observational Studies in Epidemiology (STROBE) statement (17).

### 2.1 Study population

This retrospective analysis consecutively enrolled patients who underwent the one-stop AF procedure at the Department of Cardiology, Jiangxi Provincial People's Hospital, China, between January 2021 and December 2023.

Inclusion criteria were: ① Diagnosis of non-valvular AF; ② CHA_2_DS_2_-VASc score ≥2 (for men) or ≥3 (for women); ③ Contraindication to long-term oral anticoagulation, high bleeding risk (HAS-BLED score ≥3), or strong patient preference.

Exclusion criteria were: ① Contraindications to LAAO; ② Incomplete clinical data; ③ Unavailability of any follow-up information immediately after discharge (i.e., no reachable contact and no clinical/imaging records after discharge).

Patients who became lost during longitudinal follow-up after having at least one post-discharge contact were not excluded; they were treated as censored at the time of last contact for time-to-event analyses, and a conservative worst-case scenario sensitivity analysis was additionally performed to evaluate potential outcome bias.

**Figure 1:**
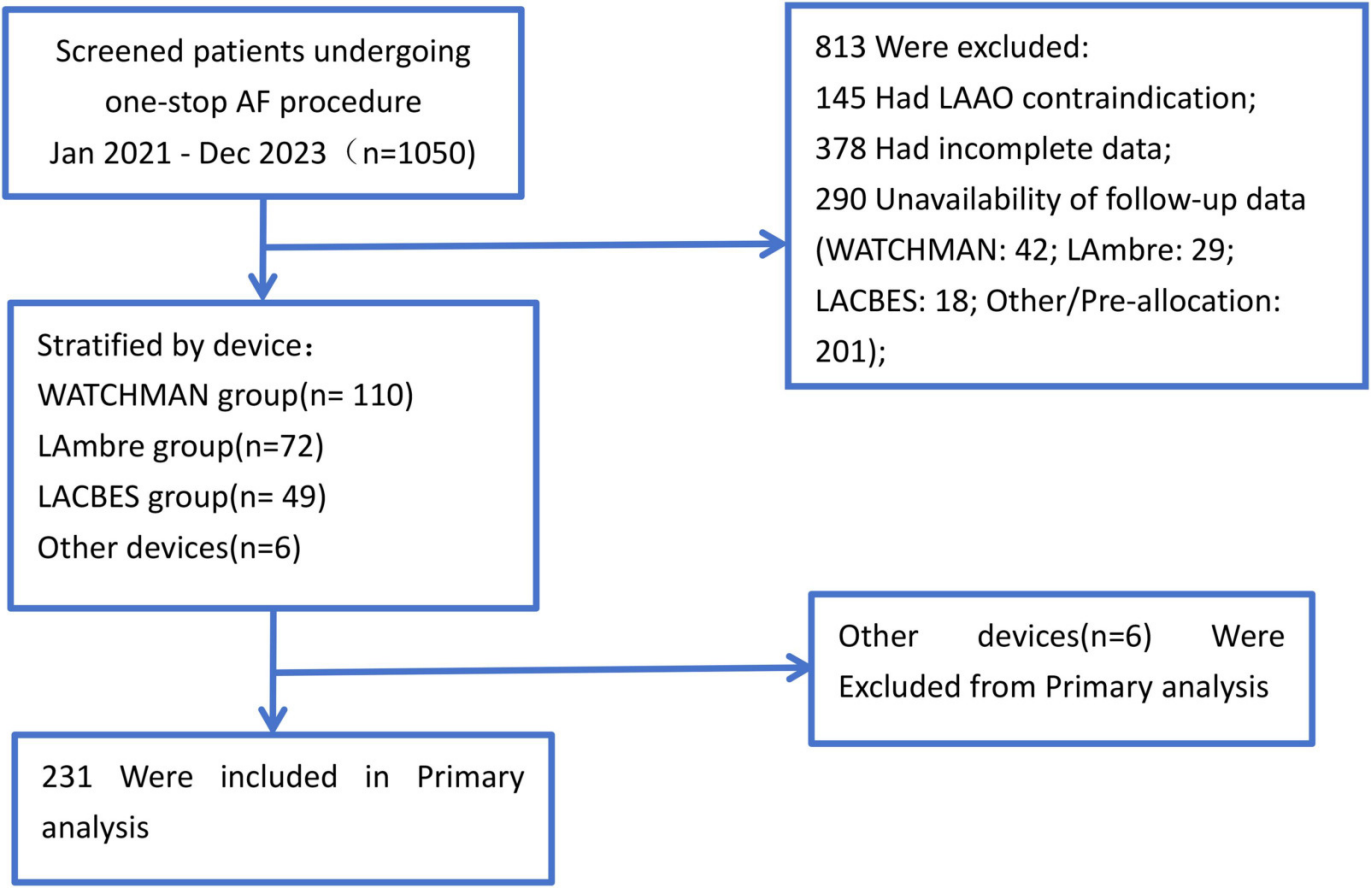
Flow chart of the study.

A total of 237 patients were initially enrolled and categorized into four groups based on the implanted device: WATCHMAN (*n* = 110), LAmbre (*n* = 72), LACBES (*n* = 49), and Other Devices (*n* = 6) (Figure 1). The “Other Devices” group (*n* = 6) was excluded from the primary analysis, resulting in a final analyzable cohort of 231 patients.

### 2.2 Procedural protocol

All patients underwent pre-procedural transesophageal echocardiography (TEE) to exclude left atrial and LAA thrombus. Under general anesthesia, catheter ablation—including circumferential pulmonary vein isolation and other necessary substrate modifications—was performed first. Following successful ablation, the selected LAA occluder was implanted under the guidance of digital subtraction angiography (DSA) and intracardiac echocardiography (ICE; AcuNav™, Biosense Webster), following standard operational procedures. Occluder type was selected in routine clinical practice rather than by randomization. The primary determinant for device selection was LAA anatomy assessed by ICE/DSA (ostium diameter, landing zone, depth, and morphology). In addition, device availability during the study period and operator experience with specific device designs were considered. All device choices were discussed and confirmed by at least two interventional cardiologists before final release. Patients' economic status and insurance coverage were not systematically recorded in the database and therefore could not be explicitly adjusted for, which may introduce residual confounding. ICE imaging criteria included clear visualization of the LAA orifice diameter, landing zone dimensions, depth, and lobe structure (e.g., windsock, chicken wing, cauliflower morphologies). Device sizing was based on ICE measurements: WATCHMAN device diameter was selected to be 20%–30% larger than the measured landing zone diameter, while the LAmbre and LACBES disc diameters were chosen to be 6–10 mm larger than the orifice diameter. Device position and stability were verified using the following multimodal criteria: DSA Assessment: Confirmation of stable device “anchor” positioning without movement in multiple angiographic views (right anterior oblique 30° + cranial 20° and left anterior oblique 40° + caudal 20°). ICE Assessment: Verification of complete coverage of the LAA orifice by the occluder disc and a device compression ratio between 15% and 25%. Peri-device leak (PDL) Assessment: Color Doppler confirmation of peri-device PDL width <3 mm. A tug test was performed before device release to assess stability, defined by slight device movement with immediate rebound to the original position. Release was performed against a threshold of “slight to moderate resistance” (approximately 200–400 g pull force) to ensure secure anchoring. All implantation steps were confirmed by at least two interventional cardiologists, each with experience in over 50 LAAO procedures.

### 2.3 Post-procedural management and follow-up

Postoperatively, all patients received a standard regimen of rivaroxaban (15 or 20 mg once daily) plus aspirin (100 mg once daily) for 45 days. After the 45-day TEE assessment, patients without major PDL (>5 mm) or DRT were transitioned to monotherapy (rivaroxaban 15/20 mg or aspirin 100 mg) for at least 6 months. The choice of monotherapy was based on the CHA_2_DS_2_-VASc score and bleeding risk, independent of the occluder type implanted. The distribution of patients receiving NOAC vs. aspirin monotherapy was comparable across the WATCHMAN, LAmbre, and LACBES groups (*P* = 0.842). Detailed dosages and distribution of antithrombotic regimens are summarized in Supplementary Table S4. Follow-up duration was defined as the time from the procedure date to the last clinical visit or the occurrence of an endpoint event (e.g., stroke, DRT).

#### 2.3.1 Standardized TEE protocol

TEE served as the primary imaging modality for assessing occlusion efficacy (PDL) and device-related complications (DRT). A unified follow-up schedule was established, with contingency plans for patients with poor TEE tolerance. Standard Schedule: All patients underwent TEE at 1, 6, and 12 months post-procedure, followed by annual TEE examinations. This schedule aligns with recommendations from the 2023 Chinese Expert Consensus on Percutaneous LAAO and the 2022 ESC Guidelines for the Management of Atrial Fibrillation, aiming for intensive short-term follow-up to detect early complications and stable long-term monitoring. Tolerance-Based Adjustment: For patients with severe esophageal disease (e.g., advanced esophagitis, esophageal varices) or poor TEE tolerance (e.g., severe gag reflex, inability to complete the exam), contrast-enhanced cardiac computed tomography (CCTA) was used as an alternative. The substitution rate and rationale were documented in the electronic case report form (eCRF). At the 6-month follow-up, TEE was the primary modality for evaluating PDL and DRT. However, 8 patients (WATCHMAN: *n* = 4; LAmbre: *n* = 2; LACBES: *n* = 2) underwent contrast-enhanced CCTA due to TEE intolerance. Although CCTA and TEE differ in modality, previous studies have demonstrated high diagnostic consistency between CCTA and TEE for LAAO follow-up, with CCTA showing superior sensitivity for minor PDLs. To ensure the reliability of the primary endpoint, a sensitivity analysis was performed by excluding these 8 patients, and the results remained consistent with the primary findings (Supplementary Table S6).

#### 2.3.2 Standardized PDL assessment

Peri-device leak (PDL) was assessed using a standardized multi-view TEE approach, as defined by the 2021 Consensus Statement on Imaging Assessment of LAA Occluders. Imaging Views: Assessment was performed in the mid-esophageal bicaval view, mid-esophageal LAA view (0°–45°), and transgastric short-axis view (90°–135°) using color Doppler flow imaging (CDFI) to detect flow across the device-tissue interface. Grading Criteria: PDL was classified based on the maximum diameter of the jet: None: jet diameter = 0 mm; Minimal: jet diameter < 3 mm (clinically insignificant, no change in antithrombotic therapy); Mild: jet diameter 3–5 mm (close monitoring, no immediate therapy change); Significant: jet diameter > 5 mm (considered “treatment failure,” requiring re-evaluation of antithrombotic therapy or interventional revision). The “<5 mm” clinical acceptability threshold is based on evidence from the PROTECT AF and AMULET IDE trials, which showed no increased stroke risk associated with PDLs <5 mm.

Inter-observer Reliability Test: To ensure consistency, two independent echocardiographers (each with over 5 years of experience in structural heart imaging), blinded to the device type, reviewed all TEE images. Inter-observer agreement was quantified using Cohen's Kappa coefficient, yielding a value of 0.87 (95% CI: 0.79–0.95, *P* < 0.001), indicating excellent agreement. Discrepancies were resolved by consensus with a third senior echocardiographer.

#### 2.3.3 Standardized stroke event adjudication

Stroke events were defined and adjudicated according to the World Health Organization (WHO) Stroke Definition (2021) and Cardiovascular Clinical Endpoint Committee (CEC) operating procedures. Stroke Definition: Acute focal neurological deficit confirmed by imaging, lasting ≥24 h (or leading to death within 24 h), caused by ischemic or hemorrhagic brain injury. Ischemic stroke required confirmation by diffusion-weighted MRI (DWI-MRI) showing restricted diffusion; hemorrhagic stroke required confirmation by CT or MRI showing cerebral hemorrhage. Independent Endpoint Adjudication Committee (EAC): A blinded EAC comprising three specialists—one cardiologist, one neurologist, and one neuroradiologist—was established to review all potential stroke events. The EAC operated independently of the research team and was blinded to patient device type and baseline data. Adjudication Process: For each reported neurological event, the EAC reviewed: (1) detailed clinical records (symptom onset, neurological exam findings, symptom duration); (2) neuroimaging reports (CT/MRI/DWI); (3) laboratory results (coagulation profile, blood glucose); and (4) vascular studies (carotid ultrasound, transcranial Doppler). Events were classified as: Confirmed Stroke: meeting WHO criteria with confirmatory imaging evidence; Probable Stroke: meeting clinical criteria but lacking immediate imaging (e.g., patient refused MRI); Non-Stroke Event: neurological deficit from non-vascular causes (e.g., hypoglycemia, seizure, brain tumor, traumatic brain injury), which were excluded from the stroke endpoint. Seven potential neurological events were reported; one was adjudicated as a “Non-Stroke” event (hypoglycemic encephalopathy) and excluded, leaving six confirmed/probable stroke events for final analysis.

### 2.4 Study endpoints

The primary efficacy endpoint was the presence of peri-device leak (PDL) at 6 months, assessed as a cross-sectional outcome at the scheduled imaging follow-up. To enhance clinical interpretability, PDL severity was stratified into three grades based on international consensus: minor (<3 mm), moderate (3–5 mm), and major (>5 mm). For the primary comparative and regression analyses, the efficacy endpoint was defined as moderate-or-greater PDL (≥3 mm), whereas minor PDL (<3 mm) was reported descriptively. As shown in Supplementary Table S5, most observed PDLs were minor (9.1% overall), and no major PDL (>5 mm) occurred in any group; the incidence of moderate-or-greater PDL (≥3 mm) did not differ significantly among the three occluders (*P* = 0.815).

The primary safety endpoints included the incidence of device-related thrombosis (DRT), thromboembolic events.

### 2.5 Statistical analysis

Statistical analyses were performed using SPSS 26.0 and R 4.1.0 software. Continuous variables are presented as mean ± standard deviation and compared using ANOVA or the Kruskal–Wallis H test, as appropriate. Categorical variables are presented as counts (percentages) and compared using the *χ*^2^ test or Fisher's exact test, the latter specifically employed due to the small expected cell counts (<5) for rare safety endpoints like DRT and stroke. To mitigate selection bias, propensity score-based inverse probability of treatment weighting (IPTW) was performed using a generalized boosted model to calculate stabilized weights. This balanced all baseline covariates across the three device groups, with success confirmed by a standardized mean difference (SMD) <0.1. The distribution of IPTW weights was inspected to ensure numerical stability and absence of extreme weights. As a robustness check, extreme weights were truncated at the 1st and 99th percentiles (if present), and weighted analyses were repeated to confirm consistency of the estimates. For binary outcome variables like PDL, where event numbers were relatively low, Firth's penalized maximum likelihood estimation was used for univariate and multivariate binary logistic regression analyses to avoid bias inherent in traditional logistic regression with sparse or separated data, calculating odds ratios (OR) with 95% confidence intervals (CI) that are inherently adjusted for penalization. PDL at 6 months was analyzed as a cross-sectional binary outcome (PDL ≥3 mm vs. <3 mm), rather than a time-to-event endpoint. Stroke-free survival was analyzed using the Kaplan–Meier method, with group comparisons made by the log-rank test. A *P*-value < 0.05 was considered statistically significant.

To evaluate potential attrition bias, a worst-case scenario analysis was performed for the stroke endpoint, where all patients lost to follow-up were conservatively treated as having experienced a stroke. Intergroup incidences were compared using a 3 × 2 contingency table with the Pearson Chi-square test.

*Post-hoc* power analysis indicated that with the present sample size (*n* = 231, event rate ≈7%), the study had 80% power (*α* = 0.05, two-sided) to detect an absolute difference of 9.8% in PDL rate between groups. However, power for stroke events (event rate ≈1.3%) was only 33%, indicating that the analysis of rare endpoints was exploratory.

Sensitivity Analysis: To address the low event count in the LACBES group (*n* = 49, 2 PDL events), LACBES and LAmbre were combined into a single “Non-WATCHMAN” group. A Firth-penalized binary logistic regression model was used to re-compare the WATCHMAN group (*n* = 110) with the Non-WATCHMAN group (*n* = 121). The *post-hoc* power for this comparison (overall event rate 6.6%, allocation ratio ≈1:1.1) was 80%, capable of detecting an 8.5% absolute difference (e.g., 4% vs. 12.5%) at *α* = 0.05 (two-sided).

### 2.6 Sample size calculation (prospective validation)

A prospective sample size calculation was performed before patient enrollment to ensure sufficient statistical power to detect clinically meaningful differences in the primary efficacy endpoint (PDL rate) among the three device groups. The calculation was based on previously published data from studies on LAAO within one-stop AF procedures, using G*Power 3.1.9.7 software (Heinrich Heine University, Düsseldorf, Germany), with preset statistical parameters consistent with standard cardiovascular device trial design.

Key Assumptions and Reference Data: Calculations were based on the following evidence-based assumptions for PDL rates: WATCHMAN group: 8.5% [consistent with long-term PROTECT AF follow-up data (14)]; LAmbre group: 6.5% [based on a multicenter LAmbre one-stop procedure study (10)]; LACBES group: 4.5% [based on a single-center preliminary LACBES study (18)]. A minimum absolute difference of 4% in PDL rate between any two groups was deemed clinically significant, based on clinical consensus linking a >4% difference to a two-fold increase in late stroke risk (8, 16). Statistical parameters were set as follows: type I error rate (*α*) = 0.05 (two-sided), power (1-*β*) = 80%, and an anticipated dropout rate of 15% [based on an average 12%–18% dropout rate in single-center retrospective studies (6, 7)].

Calculation Results and Enrollment Target: To detect the maximum anticipated absolute difference of 4% between the WATCHMAN (8.5%) and LACBES (4.5%) groups, 68 patients were required per group. Accounting for a 15% dropout rate, the total required sample size was 68 × 3 ÷ (1–0.15) ≈ 240 patients. Thus, the enrollment target was set at 240 patients, stratified as WATCHMAN: ∼80, LAmbre: ∼80, LACBES: ∼80.

Actual vs. Target Enrollment Alignment: The final analyzable cohort comprised 231 patients, representing 96.25% of the target (*n* = 240). We acknowledge that the LACBES group (*n* = 49) did not reach the preset target of 68 patients. As a newly approved domestic device (approved in China in 2020), its clinical use at our center expanded gradually during the study period, resulting in 49 enrolled patients (target 80). The actual dropout rate (12.1%) was lower than the anticipated 15%, partially compensating for the enrollment deficit.

Power Verification for Primary and Secondary Endpoints: *Post-hoc* power analysis confirmed that for the primary endpoint (PDL rate), the study retained 80.3% power (*α* = 0.05, two-sided) to detect the pre-specified 4% absolute difference in the final cohort (*n* = 231). To mitigate the potential impact of the smaller sample size in the LACBES group on the reliability of our findings, we prioritized the use of Firth's penalized-likelihood regression for multivariate analysis, a robust method specifically designed to reduce bias in smaller samples or rare event data. As anticipated in the prospective design, the study was underpowered for the rare safety endpoints: DRT (overall incidence 7.8%, power = 42.1%) and stroke (overall incidence 2.6%, power = 33.0%). This was recognized during the study design phase; given the low incidence of DRT and stroke in LAAO trials [typically <5% at 2 years (8, 14)], a single-center study would be unable to recruit sufficient patients to power these endpoints adequately. Consequently, the study was prospectively designed to focus on the primary endpoint, with secondary endpoints analyzed exploratorily.

## 3 Results

### 3.1 Comparison of baseline characteristics

No significant differences were observed among the three groups regarding age, gender, CHA_2_DS_2_-VASc score, HAS-BLED score, or the prevalence of comorbidities such as hypertension and diabetes (all *P* > 0.05), indicating comparability (Table 1). The median follow-up time for all patients was 30.5 months [interquartile range (IQR): 25.5–35.5 months]. Of the 1050 screened patients, 290 (27.6%) were excluded due to unavailability of follow-up data. No systematic documentation regarding the specific reasons for follow-up loss (e.g., patient relocation or withdrawal of consent) was available in the historical electronic case report forms, necessitating the conservative worst-case scenario sensitivity analysis. The follow-up rates were comparable across the three major device groups [WATCHMAN: 72.4% (110/152), LAmbre: 71.3% (72/101), and LACBES: 73.1% (49/67); *P* = 0.984]. The median follow-up times were 30.0 months (IQR: 25.0–35.0), 31.0 months (IQR: 26.0–36.5), and 30.0 months (IQR: 25.5–34.5) for the WATCHMAN, LAmbre, and LACBES groups, respectively, with no significant intergroup difference (*P* = 0.721).

**Table 1:** Baseline characteristics of patients by device type.

Characteristic	WATCHMAN (*n* = 110)	LAmbre (*n* = 72)	LACBES (*n* = 49)	*P*-value
Demographics				
Age, years	66.7 ± 8.8	67.9 ± 9.3	69.1 ± 7.5	0.225
Female, *n* (%)	50 (45.5)	30 (41.7)	20 (40.8)	0.589
BMI, kg/m^2^	24.9 ± 4.8	24.3 ± 5.4	22.5 ± 4.7	0.076
Clinical Scores				
CHA_2_DS_2_-VASc	4.0 ± 1.4	3.7 ± 1.4	4.2 ± 1.4	0.264
HAS-BLED	1.4 ± 1.0	1.4 ± 0.9	1.6 ± 1.0	0.737
Comorbidities, *n* (%)				
Hypertension	69 (62.7)	39 (54.2)	31 (63.3)	0.756
Diabetes	25 (22.7)	8 (11.1)	10 (20.4)	0.337
Heart failure	64 (58.2)	43 (59.7)	24 (49.0)	0.225
Stroke/TIA history	50 (45.5)	26 (36.1)	22 (44.9)	0.431
Renal dysfunction	4 (3.6)	4 (5.6)	4 (8.2)	0.107
AF Type, *n* (%)				
Paroxysmal AF	26 (23.6)	13 (18.1)	10 (20.4)	0.440
Persistent AF	84 (76.4)	58 (80.6)	39 (79.6)	
Permanent AF	0 (0.0)	1 (1.4)	0 (0.0)	

Data presented as mean ± standard deviation or *n* (%). *P*-values calculated using ANOVA for continuous variables and chi-square test for categorical variables.

### 3.2 Occlusion efficacy and device-related thrombosis

PDL rates were 8.2% (9/110), 6.9% (5/72), and 4.1% (2/49) for the WATCHMAN, LAmbre, and LACBES groups, respectively, showing no statistically significant difference (*P* > 0.05). DRT incidence also did not differ significantly among the groups (Table 2).

**Table 2:** Comparison of clinical outcomes by device type.

Characteristic	WATCHMAN (*n* = 110)	LAmbre (*n* = 72)	LACBES (*n* = 49)	*P*-value
PDL, *n* (%)	9 (8.2)	5 (6.9)	2 (4.1)	0.381
Device-related thrombosis, *n* (%)	7 (6.3)	9 (12.4)	2 (4.1)	0.220
Stroke events, *n* (%)	2 (1.8)	4 (5.6)	1 (2.9)	0.321

Firth Regression Analysis for Safety Endpoints				
Outcome	Variable	OR	95% CI	*P*-value
Device-related thrombosis				
LAmbre vs WATCHMAN	1.31	0.01–242.73	0.894	
LACBES vs. WATCHMAN	7.05	0.37–1,031.70	0.189	
CHA_2_DS_2_-VASc score	0.72	0.11–2.17	0.606	
Stroke events				
LAmbre vs. WATCHMAN	1.75	0.26–12.04	0.549	
LACBES vs. WATCHMAN	0.40	0.00–5.11	0.523	
CHA_2_DS_2_-VASc score	0.95	0.46–1.88	0.895	

Data are presented as *n* (%). *P*-values were calculated using the chi-square test for PDL and Fisher's exact test for device-related thrombosis and stroke events. Fisher' s exact test was employed for DRT and stroke events due to the small expected cell counts (<5) for these rare safety outcomes.

### 3.3 Multivariate analysis of factors influencing PDL: firth correction results

Variables with *P* < 0.1 in univariate analysis, along with device type, were included in the multivariate analysis. To overcome estimation instability due to the small number of events, a Firth-corrected logistic regression model was employed. After adjusting for CHA_2_DS_2_-VASc score, bleeding history, age, and gender, device type was not an independent predictor of PDL (LAmbre vs. WATCHMAN: OR = 0.99, 95% CI: 0.30–2.97, *P* = 0.986; LACBES vs. WATCHMAN: OR = 0.62, 95% CI: 0.11–2.35, *P* = 0.500). Conversely, a higher CHA_2_DS_2_-VASc score was an independent risk factor (OR = 1.67, 95% CI: 1.12–2.52, *P* = 0.011), indicating a 67% increase in PDL risk per 1-point score increase. Older age emerged as a protective factor (OR = 0.94, 95% CI: 0.89–1.00, *P* = 0.035), suggesting a 6% decrease in risk per additional year of age. Bleeding history and gender showed no statistically significant association with PDL (Table 3).

**Table 3:** Multivariate firth logistic regression analysis for PDL.

Variable	OR	95% CI	*P*-value
Device type (ref: WATCHMAN)			
LAmbre	0.99	0.30–2.97	0.986
LACBES	0.62	0.11–2.35	0.500
Other	0.70	0.00–8.46	0.817
CHA_2_DS_2_-VASc score	1.67	1.12–2.52	0.011
Age (per year)	0.94	0.89–1.00	0.035
Bleeding history	0.84	0.09–3.86	0.847
Female gender	1.00	0.35–2.86	0.995

OR, odds ratio; CI, confidence interval. Analysis performed using Firth's penalized maximum likelihood estimation to address rare events. The 95% confidence intervals (CIs) derived from Firth's penalized likelihood regression are adjusted for penalization.

### 3.4 Stroke events and survival analysis

**Figure 2:**
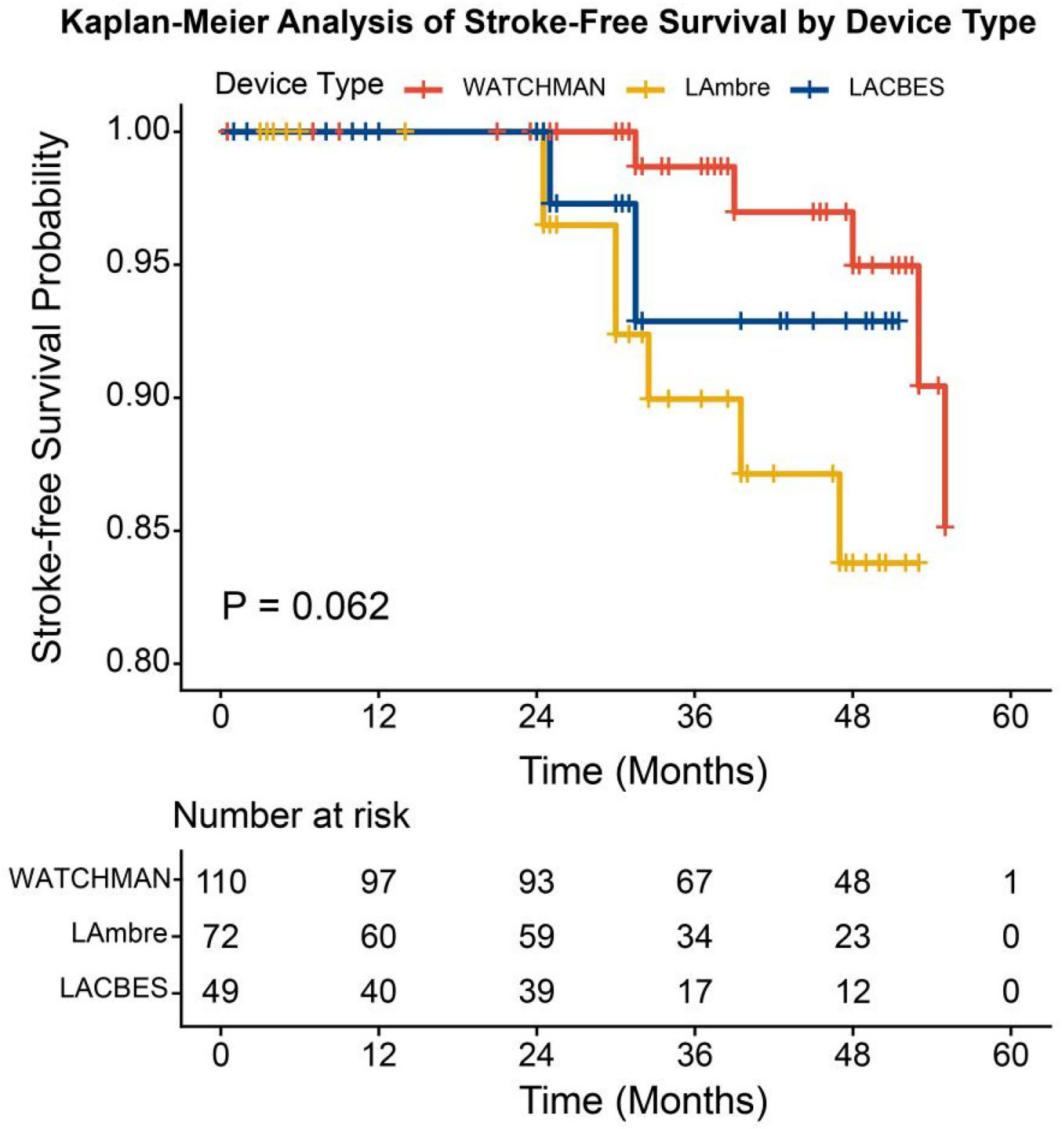
Kaplan–Meier curves for stroke-free survival. No significant difference was observed among patients implanted with WATCHMAN, LAmbre, or LACBES occluders during the follow-up period (Log-rank *P* = 0.062). The table below the curves shows the number of patients at risk at each time point.

No significant differences were found in stroke incidence among the three groups (Table 2). Kaplan–Meier curves revealed no significant differences in stroke-free survival (Log-rank *P* > 0.05) (Figure  2). Visually, the Kaplan–Meier curves for stroke-free survival largely overlapped across all three device groups, demonstrating no meaningful separation over the 50-month follow-up window, which aligned with the non-significant log-rank result. After adjustment using the Cox proportional hazards model, no significant association was found between device type and stroke risk.

**Figure 3:**
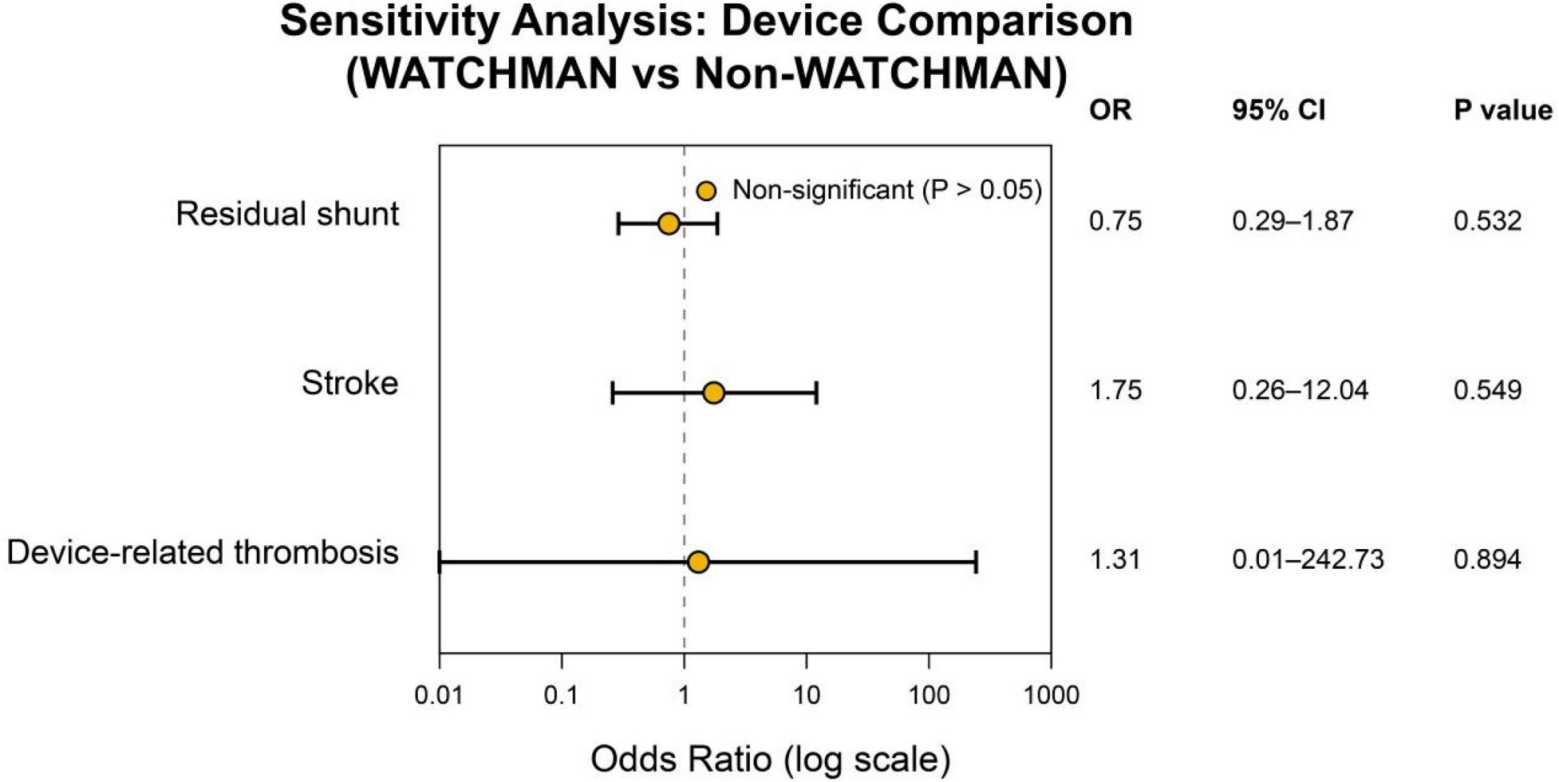
Forest plot of sensitivity analysis comparing outcomes between WATCHMAN and Non-WATCHMAN devices (LAmbre and LACBES combined). Odds ratios (OR) with 95% confidence intervals (CI) are shown for residual shunt (PDL ≥3mm), stroke, and device-related thrombosis. No significant differences were observed between the two groups for any outcome (all *P*>0.05). The analysis was performed using Firth's penalized likelihood regression to account for low event rates.

### 3.5 Sensitivity analyses

#### 3.5.1 Propensity score weighting (IPTW)

To address potential selection bias, outcomes were re-evaluated after IPTW adjustment. As demonstrated in Supplementary Table S1, all baseline covariates achieved an optimal balance after weighting (all SMDs < 0.1). Weighted Firth-corrected logistic regression confirmed the primary findings, showing that device type remained a non-significant predictor of PDL (Supplementary Table S2; LAmbre vs. WATCHMAN: Adjusted OR = 1.02, 95% CI: 0.35–2.88, *P* = 0.965; LACBES vs. WATCHMAN: Adjusted OR = 0.68, 95% CI: 0.15–2.42, *P* = 0.540; LACBES vs. LAmbre: Adjusted OR = 0.67, *P* = 0.612).

#### 3.5.2 Worst-case scenario analysis for stroke events

To evaluate whether missing follow-up data for 89 screened patients (WATCHMAN: *n* = 42; LAmbre: *n* = 29; LACBES: *n* = 18) could have biased the safety comparisons, a worst-case scenario analysis was conducted. Reasons for missing follow-up data were not systematically documented in the eCRF; therefore, we conservatively assumed that every patient with missing follow-up experienced a stroke event to assess the maximum potential impact of attrition bias (Supplementary Table S3). Under this extreme assumption, the simulated incidences were 40.0% (44/110) for WATCHMAN, 45.8% (33/72) for LAmbre, and 38.8% (19/49) for LACBES. The 3 × 2 contingency table analysis revealed no significant intergroup difference even in this highly conservative scenario (*χ*^2^ = 0.808, *P* = 0.668), suggesting that our primary conclusions regarding comparable device safety were unlikely to be materially driven by missing follow-up data.

#### 3.5.3 Robustness of primary findings

To verify the robustness of the primary findings, a sensitivity analysis was conducted by merging the LAmbre and LACBES groups into a “Non-WATCHMAN” group (comprising LAmbre and LACBES devices) for comparison. Firth penalized-likelihood regression showed no significant difference in PDL incidence between the WATCHMAN (*n* = 110) and Non-WATCHMAN (*n* = 121) groups (adjusted OR = 0.75, 95% CI: 0.29–1.87, *P* = 0.532). Similarly, no significant differences were observed for stroke events (adjusted OR = 1.75, 95% CI: 0.26–12.04, *P* = 0.549) or DRT incidence (adjusted OR = 1.31, 95% CI: 0.01–242.73, *P* = 0.894). Goodness-of-fit tests indicated adequate model fit (Likelihood ratio test *χ*^2^ = 1.98, df = 4, *P* = 0.739) (Table 4).

**Table 4:** Sensitivity analysis: comparison of clinical outcomes between WATCHMAN and Non-WATCHMAN devices.

Outcome	WATCHMAN (*n* = 110)	Non-WATCHMAN^a^ (*n* = 121)	Adjusted OR (95% CI)^b^	*P* Value
PDL	9 (8.2%)	7 (5.8%)	0.75 (0.29–1.87)	0.532
Stroke	2 (1.8%)	5 (4.1%)	1.75 (0.26–12.04)	0.549
Device-related thrombosis	7 (6.4%)	11 (9.1%)	1.31 (0.01–242.73)	0.894

a Non-WATCHMAN group includes LAmbre (*n* = 72) and LACBES (*n* = 49) devices.

b Odds ratios adjusted for CHA_2_DS_2_-VASc score, age, and bleeding history using Firth's penalized likelihood regression. Model fit: likelihood ratio test *χ*^2^ = 1.98, df = 4, *P* = 0.739. The 95% confidence intervals (CIs) derived from Firth's penalized likelihood regression are adjusted for penalization.

*Post-hoc* Bayesian power analysis indicated 78% Bayesian power (95% credible interval lower limit 63%) for the PDL endpoint when the true difference was ≥3%. For the stroke endpoint, Bayesian power was only 31% (95% credible interval lower limit 18%), confirming the exploratory nature of the analysis for rare safety events.

Three additional sensitivity analyses were performed:

Exclusion of patients with follow-up <12 months (*n* = 28): Analysis of the remaining 203 patients showed that PDL rates, DRT, and stroke incidence remained comparable across groups. Multivariate analysis conclusions were consistent with the primary analysis (Table 5).

**Table 5:** Outcomes after excluding patients with follow-up <12 months.

Outcome	WATCHMAN (*n* = 92)	LAmbre (*n* = 61)	LACBES (*n* = 50)	*P*-value
PDL, *n* (%)	7 (7.6%)	4 (6.6%)	2 (4.0%)	0.412
DRT, *n* (%)	5 (5.4%)	7 (11.5%)	3 (6.0%)	0.287
Stroke, *n* (%)	1 (1.1%)	3 (4.9%)	1 (2.0%)	0.358
DRT, device-related thrombosis.				

Multivariate logistic regression analysis for PDL.			
Variable	Odds Ratio (OR)	95% Confidence Interval	*P*-value
Device type (ref: WATCHMAN)			
LAmbre	1.02	0.29–3.57	0.978
LACBES	0.58	0.10–3.26	0.539
CHA_2_DS_2_-VASc score	1.71	1.13–2.59	0.010
Age	0.93	0.88–0.99	0.031

The model was adjusted for device type, CHA_2_DS_2_-VASc score, and age.

Subgroup analysis stratified by CHA_2_DS_2_-VASc score (cut-off 5): No significant difference in PDL risk between Non-WATCHMAN and WATCHMAN devices was found in either CHA_2_DS_2_-VASc ≤5 (*n* = 160) or >5 (*n* = 77) subgroups. The interaction test was non-significant (*P* for interaction = 0.661), indicating no significant effect modification by stroke risk score (Table 6).

**Table 6:** Subgroup analysis of PDL risk.

Subgroup	Patients, n	Non-WATCHMAN events, n/N	WATCHMAN events, n/N	Odds ratio (95% CI)	*P*-value	P for interaction
All patients	231	7/121	9/110	0.75 (0.29–1.87)	0.532	-
CHA_2_DS_2_-VASc						0.661
≤5	154	3/74	4/80	1.35 (0.29–6.24)	0.700	
>5	77	4/47	5/30	2.15 (0.53–8.75)	0.285	
Age						0.390
>65 years	152	6/77	6/75	0.88 (0.20–3.42)	0.851	
≤65 years	79	1/38	3/41	2.26 (0.54–12.97)	0.276	

CI, confidence interval. The Non-WATCHMAN group includes LAmbre and LACBES devices. Odds ratios and *P*-values were derived from Firth's penalized likelihood regression models, adjusted for relevant covariates. The 95% confidence intervals (CIs) derived from Firth's penalized likelihood regression are adjusted for penalization.

#### 3.5.4 Subgroup analyses

Subgroup analysis stratified by age (cut-off 65 years): No significant difference in PDL risk was observed between Non-WATCHMAN and WATCHMAN devices in either the >65 years (*n* = 158) or ≤65 years (*n* = 79) subgroups. Interaction analysis indicated that age was not a significant effect modifier (Table 6).

## 4 Discussion

This study is the first to apply Firth's penalized maximum likelihood estimation in comparing different occluders within the one-stop AF procedure, effectively mitigating the estimation bias and abnormally wide confidence intervals associated with traditional logistic regression when handling low-frequency event data like PDL, thereby ensuring the robustness of the findings.

The central finding of this study is that the WATCHMAN, LAmbre, and LACBES occluders—despite their divergent designs—demonstrated comparable sealing efficacy and safety profiles in the context of the one-stop AF procedure. Notably, the comparable performance of the domestically developed LACBES occluder merits specific discussion. As a relatively new entrant compared to the established WATCHMAN and LAmbre devices, the LACBES device exhibited a numerically lowest PDL rate (4.1% vs. 8.2% and 6.9%) and a favorable DRT profile (4.1% vs. 6.3% and 12.4%) in our cohort, albeit without statistical significance. The LACBES device shares a dual-disc design philosophy with the LAmbre occluder, intended to better accommodate complex LAA anatomies (19). However, its distinctive features include a potentially broader disc contact area and a unique anchoring mechanism. Our findings suggest that within the optimized environment of the one-stop procedure, this design is equally capable of achieving stable and effective sealing. The success of LACBES underscores that the benefits of the one-stop strategy may extend to a broader range of device designs, including those developed domestically. This is particularly significant for healthcare systems considering cost-effectiveness and device accessibility. The promising results with LACBES indicate that it represents a viable and effective alternative to imported devices in the one-stop procedure context. Future studies with larger sample sizes and longer follow-up are warranted to further validate its long-term safety and to explore whether its specific design features confer particular advantages in certain anatomical subsets, especially within the synergistic framework of combined CA and LAAO. This observation contrasts with trends of device-dependent differences reported in some studies involving standalone LAAO (8, 19), suggesting the possibility that the one-stop procedural paradigm, through its unique synergistic effects, may reduce the dependency on specific device designs, resulting in a “homogenized” occlusion outcome. We did not perform a formal cost-effectiveness analysis because detailed cost and reimbursement data were not systematically captured; this remains an important direction for future research, particularly when considering domestically manufactured devices in resource-limited settings.

### 4.1 Does the one-stop procedure alter device compatibility requirements?

Our findings are consistent with, but do not directly prove, the hypothesis that preceding CA may create a more favorable implantation milieu for LAAO. In standalone LAAO, complex LAA anatomy (e.g., cauliflower or multi-lobed shapes) often dictates the choice of a double-disc device like LAmbre for its potential to better adapt to irregular orifices. However, in this study, comparable sealing success was achieved across all three devices despite the patient population encompassing various LAA morphologies. We hypothesize that preceding CA in the one-stop setting optimizes the implantation environment through two key mechanisms: Acute Hemodynamic Improvement: Terminating AF and restoring sinus rhythm immediately reduces left atrial pressure and LAA blood stasis, diminishing the interference of “flow” on initial device apposition; Long-Term Reverse Remodeling: Successful ablation holds the potential to reverse aspects of atrial electrophysiological and structural remodeling (5, 20, 21), potentially leading to a more stable LAA orifice and reduced contractility over time. This “calmer” LAA environment may mitigate the stringent compatibility demands imposed by complex anatomy, allowing various device designs to achieve stable closure from a more favorable baseline. In other words, the one-stop procedure may broaden the effective compatibility range of different occluder types.

### 4.2 Potential synergistic effects of the one-stop procedure: a hypothesis-generating perspective

It is important to acknowledge that this study lacked a direct standalone LAAO control group, and our patient population—including stroke risk scores and comorbidity profiles—was not matched with those in historical registries. Consequently, the observation that our overall PDL rate (6.9%) and numerical DRT incidence appear lower than some large-scale standalone LAAO (8, 19) registries remains purely exploratory and cannot substantiate a claim of clinical superiority for the one-stop procedure. Nevertheless, based on the potential “synergistic effect” mechanism—where catheter ablation terminates AF and restores sinus rhythm, potentially optimizing the implantation environment—we propose a hypothesis for future validation: for the same patient, occluder implantation within a one-stop procedure may yield a higher probability of a perfect seal (PDL <3 mm) and a potentially lower risk of DRT. This hypothesis suggests that the one-stop strategy warrants further investigation through randomized, matched-pair studies to determine whether it truly optimizes therapeutic outcomes of LAA occlusion beyond procedural convenience.

Our results align with the recent trend emphasizing the comprehensive value of the one-stop procedure. For instance, the OPTION randomized trial evaluated LAAO after AF ablation as an alternative to oral anticoagulation in patients with atrial fibrillation (7). In the final OPTION results, LAAO after AF ablation was shown to be noninferior to oral anticoagulation for the composite efficacy endpoint of all-cause death, stroke, or systemic embolism at 36 months, rather than demonstrating a significant reduction in stroke or systemic embolism alone (5). Our study adds a “device selection” perspective to these macro-level conclusions: within the proven clinically beneficial one-stop paradigm, operators can flexibly choose from a range of effective tools, including domestic devices, based on anatomical suitability, device availability, and cost-effectiveness, without excessive concern over the absolute superiority of one design's efficacy. This reinforces the feasibility of the one-stop strategy as an integrated, individualized solution.

A key finding of this study is the identification of the CHA_2_DS_2_-VASc score as an independent risk factor for post-procedural PDL. This likely reflects the greater burden of AF and the severity of atrial cardiomyopathy associated with high scores (22, 23). These factors collectively promote more pronounced structural remodeling of the left atrium and LAA (24–27), complicating complete endothelial apposition of the occluder. Even with a seemingly “perfect” implant, the persistent cyclic cardiac motion over time might induce micro-gaps, manifesting as PDL. Therefore, the CHA_2_DS_2_-VASc score can serve as a practical clinical tool for identifying the “complex LAA”. Given the lack of significant efficacy differences among the three occluders, device selection should prioritize anatomical suitability. For instance, WATCHMAN's radial force may be preferred for regular orifices, LAmbre's dual-disc design might offer better stability for irregular openings, and LACBES presents a viable alternative for moderately remodeled LAAs, considering cost or availability. Enhanced post-procedural TEE monitoring (e.g., first TEE at 3 months, then every 6 months for the first 2 years) is recommended for high-score patients.

Counterintuitively, this study found that increasing age was a protective factor against PDL. A plausible explanation is that age-related progression of atrial fibrosis significantly reduces the LAA's active contractile function and elasticity (28–33). A “stiffer” appendage offers less resistance to the persistent radial force of the occluder, potentially leading to more stable and enduring tissue-device apposition. Conversely, the stronger atrial myocardial contractility and greater elasticity/mobility of the LAA in younger patients subject the device interface to continuous “flow” and “traction” forces, theoretically increasing the risk of micro-displacement or malapposition, thus leading to PDL. This finding, seemingly contradictory to the “high CHA_2_DS_2_-VASc score as a risk factor,” actually reveals the duality of PDL risk: “complex anatomy” on one hand, and a “highly dynamic environment” in younger hearts on the other. Future studies should consider both mechanisms. For younger patients, device selection favoring long-term stability (e.g., WATCHMAN's embolization design or LACBES's broader disc contact) and intensified early and long-term imaging follow-up are advisable.

Regarding safety, a noteworthy trend observed in this study was the numerically higher incidence of DRT with double-disc devices (LAmbre 12.4% and LACBES 4.1%) compared to the plug-based WATCHMAN device (6.3%). This finding echoes the results of the landmark AMULET IDE trial, which also reported a higher DRT incidence with the Amulet occluder compared to WATCHMAN 2.5 (8). However, a crucial distinction in our one-stop procedure cohort is that this numerical difference did not translate into a significant increase in stroke events nor reach statistical significance. We speculate that this may reveal a potential modifying effect of the one-stop procedure: the maintenance of sinus rhythm post-ablation, by improving left atrial hemodynamics and promoting faster, more uniform endothelialization, might partially counteract the inherent differences in DRT risk propensity among different device designs. This indicates that the context for evaluating device safety may have shifted from “standalone LAAO” to the “one-stop procedure”. The underlying reasons for the numerical DRT rate differences may relate to design-dependent endothelialization processes; WATCHMAN's porous polyester cover may provide a superior scaffold for rapid endothelial cell overgrowth, whereas the central suture zone or bare waist of double-disc devices might present a relatively prolonged “endothelialization window,” theoretically increasing thrombotic risk during this period. However, given the limited statistical power for rare safety endpoints in our cohort (*post-hoc* power: DRT 42.1% and stroke 33.0%), these observations should be interpreted as hypothesis-generating rather than definitive evidence of device-related safety differences.

### 4.3 Limitations

The interpretation of our results must be contextualized within the following limitations, which we endeavored to mitigate through corresponding statistical design:

This cannot fully exclude the influence of unmeasured confounders, particularly detailed LAA anatomical variables (e.g., ostium/landing-zone dimensions, depth, and morphology), which were not systematically captured in a structured dataset for all patients and therefore could not be incorporated into adjustment models, as well as operator preference in device selection, potentially introducing selection bias. Accordingly, we were unable to perform stratified or sensitivity analyses by LAA morphology or device size, and residual confounding related to anatomy may persist. However, we observed no statistically significant differences in measured baseline clinical characteristics across the three groups (Table 1) and consistently adjusted for known key confounding factors in all multivariate models, thereby enhancing the comparability and credibility of the results to a reasonable extent. To further reduce selection bias from measured covariates, we implemented propensity score–based inverse probability of treatment weighting (IPTW), achieving good balance across device groups (all weighted SMDs <0.1; Supplementary Table S1). Nevertheless, IPTW cannot account for unmeasured confounders (e.g., patient socioeconomic status/insurance coverage and operator preference), which remains an inherent limitation. In addition, as this was a single-center experience, device allocation may reflect local availability and institutional practice patterns, and operator learning curves and center-level workflows may limit generalizability.

Second, the imbalance in group sizes (particularly the smaller LACBES group) and the inadequate statistical power for rare safety endpoints (like DRT and stroke) limit our ability to draw definitive conclusions regarding long-term safety differences among devices. This was explicitly acknowledged in both *a priori* and *post-hoc* power analyses. But precisely because we anticipated this limitation, the study's design focus was consistently directed at the primary endpoint (PDL rate), for which it was adequately powered. More importantly, we provided a *post-hoc* Bayesian power analysis (Section 3.5) to transparently report the evidentiary strength, which confirmed the exploratory nature of these safety findings. Furthermore, to verify that our safety conclusions were not biased by the exclusion of missing data or low event rates, we conducted a worst-case scenario analysis (Supplementary Table S3). This extreme-case simulation demonstrated that even if all lost-to-follow-up patients were assumed to have experienced a stroke, the intergroup differences remained non-significant. Combined with the use of Firth-corrected logistic regression, these strategies ensure that our findings, while exploratory, are statistically stable and robust against small-sample bias.

Third, mechanistic explanations regarding hemodynamic improvement and LAA functional changes after CA (e.g., left atrial pressure, LAA emptying velocity/fraction) were not directly measured in this retrospective cohort. Therefore, the proposed “synergistic effect” should be regarded as hypothesis-generating and warrants prospective validation with dedicated physiological assessments.

Finally, the median follow-up of 30.5 months represents a mid-term assessment and may be insufficient to fully capture very late complications, such as late-onset DRT or occluder migration. Nonetheless, this study incorporated a standardized imaging follow-up protocol covering the early to mid-term period, ensuring adequate capture of most typical early and mid-term complications (like significant PDL and early DRT).

In summary, we openly acknowledge these limitations but emphasize the implementation of the most appropriate statistical strategies currently available within the study's constraints to address them. Consequently, we believe the conclusion regarding the comparable mid-term occlusion efficacy of the three devices in the one-stop procedure is robust. In contrast, the safety findings should be regarded as hypothesis-generating, exploratory data, providing crucial preliminary evidence to inform subsequent larger-scale, prospective studies with longer follow-up durations.

## 5 Conclusion

This study provides preliminary evidence that within the synergistic framework of the one-stop AF procedure, the WATCHMAN, LAmbre, and LACBES occluders all achieve effective and safe LAA closure, with no significant differences in performance. This finding strongly suggests that within the one-stop context, the focus of device selection decision-making can shift from “which design is absolutely superior” to “which device is best suited to the specific anatomy of the patient and the operator's experience at a given center”. This study is the first to propose the hypothesis that the synergistic effect of the one-stop procedure may homogenize occluder performance and preliminarily elucidate its potential mechanisms. Future research, particularly randomized controlled trials directly comparing “one-stop LAAO” vs. “standalone LAAO,” should focus on validating this hypothesis and further identifying which patient subgroups derive the greatest benefit from this synergistic model, ultimately paving the way for the precise, synergistic optimization of procedural strategy and device choice. Patients with high CHA_2_DS_2_-VASc scores and those who are relatively younger may possess a higher risk of PDL and warrant intensified imaging surveillance post-procedure.

## Data Availability

The raw data supporting the conclusions of this article will be made available by the authors, without undue reservation.
