# Supplementary material for: Comparative study on the efficacy and safety of different left atrial appendage occluders in one-stop atrial fibrillation procedures
Source: Front Cardiovasc Med. 2026 May 26;13:1742519. doi: 10.3389/fcvm.2026.1742519 (PMC13246385; doi:10.3389/fcvm.2026.1742519)
Supplement: Supplementary file 1 [file Datasheet1.docx]

| Table S1: Baseline Characteristics Balance Before and After Inverse Probability of Treatment Weighting (IPTW) | | | | | | |
| --- | --- | --- | --- | --- | --- | --- |
| Covariates | Unweighted SMD | | | Weighted (IPTW) SMD | | |
|  | LAmbre vs. WATCHMAN | LACBES vs. WATCHMAN | LACBES vs LAmbre | LAmbre vs. WATCHMAN | LACBES vs. WATCHMAN | LACBES vs LAmbre |
| **Demographics** |  |  |  |  |  |  |
| Age, years | 0.132 | 0.294 | 0.162 | 0.045 | 0.082 | 0.048 |
| Female gender | 0.077 | 0.095 | 0.021 | 0.021 | 0.038 | 0.019 |
| BMI, kg/m² | 0.118 | 0.504 | 0.386 | 0.036 | 0.091 | 0.055 |
| Clinical Scores |  |  |  |  |  |  |
| CHA₂DS₂-VASc | 0.214 | 0.143 | 0.071 | 0.052 | 0.041 | 0.028 |
| HAS-BLED | 0 | 0.2 | 0.2 | 0.015 | 0.063 | 0.048 |
| **Comorbidities** |  |  |  |  |  |  |
| Hypertension | 0.174 | 0.012 | 0.162 | 0.061 | 0.005 | 0.056 |
| Diabetes | 0.315 | 0.056 | 0.259 | 0.088 | 0.024 | 0.064 |
| Heart failure | 0.03 | 0.185 | 0.155 | 0.012 | 0.055 | 0.043 |
| Stroke/TIA history | 0.192 | 0.012 | 0.18 | 0.074 | 0.008 | 0.066 |
| Renal dysfunction | 0.096 | 0.198 | 0.102 | 0.033 | 0.076 | 0.048 |
| **AF Type** |  |  |  |  |  |  |
| Paroxysmal AF | 0.136 | 0.079 | 0.057 | 0.041 | 0.027 | 0.021 |
| Persistent/Permanent AF | 0.103 | 0.081 | 0.022 | 0.035 | 0.022 | 0.018 |

| Table S2: Clinical Outcomes After IPTW Adjustment | | | | |
| --- | --- | --- | --- | --- |
| Outcome | Comparison | Weighted Odds Ratio (OR)* | 95% Confidence Interval (CI) | P-value |
| Residual Shunt | LAmbre vs. WATCHMAN | 1.02 | 0.35 – 2.88 | 0.965 |
|  | LACBES vs. WATCHMAN | 0.68 | 0.15 – 2.42 | 0.54 |
|  | LACBES vs. LAmbre | 0.67 | 0.12 – 3.45 | 0.612 |
| Device-Related Thrombosis (DRT) | LAmbre vs. WATCHMAN | 1.45 | 0.42 – 4.96 | 0.552 |
|  | LACBES vs. WATCHMAN | 0.82 | 0.18 – 3.75 | 0.796 |
|  | LACBES vs. LAmbre | 0.57 | 0.09 – 4.10 | 0.584 |
| Stroke Events | LAmbre vs. WATCHMAN | 1.88 | 0.31 – 11.24 | 0.49 |
|  | LACBES vs. WATCHMAN | 0.55 | 0.04 – 6.81 | 0.642 |
|  | LACBES vs. LAmbre | 0.29 | 0.02 – 5.12 | 0.365 |

| Table S3: Worst-case Scenario Sensitivity Analysis for Stroke Events | | | | | |
| --- | --- | --- | --- | --- | --- |
| Device Group | Observed Stroke Events, n (%) | Patients Lost to Follow-up (LTFU), n | Worst-case Total Events, n | Total Population (N) | Worst-case Incidence, % |
| WATCHMAN | 2 (1.8%) | 42 | 44 | 110 | 40.00% |
| LAmbre | 4 (5.6%) | 29 | 33 | 72 | 45.80% |
| LACBES | 1 (2.0%) | 18 | 19 | 49 | 38.80% |
| Total | 7 (3.0%) | 89 | 96 | 231 | 41.60% |
| χ2 Value | - | - | - | - | 0.808 |
| P-value | - | - | - | - | 0.668 |

| Table S4: Distribution of Post-Procedural Antithrombotic Regimens Across Device Groups | | | | | |
| --- | --- | --- | --- | --- | --- |
| Antithrombotic Regimen Phase | Total (N=231) | WATCHMAN (n=110) | LAmbre (n=72) | LACBES (n=49) | P-value* |
| **Phase 1: Initial 45 Days (Standard)** |  |  |  |  |  |
| Rivaroxaban (15/20 mg) + Aspirin (100 mg) | 231 (100%) | 110 (100%) | 72 (100%) | 49 (100%) | - |
| Other specific regimens (e.g., OAC only due to bleeding risk) | 0 (0.0%) | 0 (0.0%) | 0 (0.0%) | 0 (0.0%) | - |
| **Phase 2: After 45-Day TEE Assessment (Monotherapy)** |  |  |  |  | 0.842 |
| Rivaroxaban Monotherapy (15 or 20 mg/day) | 148 (64.1%) | 72 (65.5%) | 45 (62.5%) | 31 (63.3%) |  |
| Aspirin Monotherapy (100 mg/day) | 78 (33.8%) | 36 (32.7%) | 25 (34.7%) | 17 (34.7%) |  |
| Continued Dual Therapy (due to DRT or severe residual shunt) | 5 (2.1%) | 2 (1.8%) | 2 (2.8%) | 1 (2.0%) |  |
| **Monotherapy Selection Criteria (Phase 2)** |  |  |  |  |  |
| High Bleeding Risk (HAS-BLED ≥ 3) switched to Aspirin | 70/78 | 32/36 | 23/25 | 15/17 | 0.915 |
| High Stroke Risk (CHA2​DS2​−VASc≥4) kept on Rivaroxaban | 135/148 | 65/72 | 41/45 | 29/31 | 0.774 |
| TEE: Transesophageal Echocardiography; NOAC: Non-vitamin K antagonist oral anticoagulant; DRT: Device-Related Thrombosis | | | | | |

| Table S5: Efficacy and Safety Endpoints at 6-Month Follow-up | | | | |
| --- | --- | --- | --- | --- |
| Endpoints | WATCHMAN (n=110) | LAmbre (n=72) | LACBES (n=49) | P-value |
| **Primary Efficacy Endpoint** |  |  |  |  |
| Overall PDL, n (%) | 9 (8.2%) | 5 (6.9%) | 2 (4.1%) | 0.655 |
| PDL Stratification: |  |  |  |  |
| • Minor (< 3 mm) | 7 (6.4%) | 4 (5.6%) | 2 (4.1%) | 0.852 |
| • Moderate (3–5 mm) | 2 (1.8%) | 1 (1.4%) | 0 (0.0%) | 0.815 |
| • Major (> 5 mm) | 0 (0.0%) | 0 (0.0%) | 0 (0.0%) | >0.999 |
| **Primary Safety Endpoints** |  |  |  |  |
| Device-Related Thrombosis (DRT) | 1 (0.9%) | 1 (1.4%) | 0 (0.0%) | 0.835 |
| Thromboembolic Events (Stroke/TIA) | 1 (0.9%) | 2 (2.8%) | 0 (0.0%) | 0.462 |
| AF: Atrial Fibrillation; BARC: Bleeding Academic Research Consortium; TIA: Transient Ischemic Attack. | | | | |

| Table S6: Sensitivity Analysis of 6-Month Imaging Endpoints Excluding Patients Followed by CCTA | | | | |
| --- | --- | --- | --- | --- |
| Analysis Set & Endpoints | WATCHMAN | LAmbre | LACBES | P-value |
| Primary Analysis (Full Cohort) | n=110 | n=72 | n=49 |  |
| Overall PDL (≥3 mm), n (%) | 9 (8.2%) | 5 (6.9%) | 2 (4.1%) | 0.643 |
| Device-Related Thrombosis (DRT), n (%) | 1 (0.9%) | 1 (1.4%) | 0 (0.0%) | 0.835 |
| Sensitivity Analysis (Excluding CCTA)† | n=106 | n=70 | n=47 |  |
| Overall PDL (≥3 mm), n (%) | 9 (8.5%) | 5 (7.1%) | 2 (4.3%) | 0.645 |
| Device-Related Thrombosis (DRT), n (%) | 1 (0.9%) | 1 (1.4%) | 0 (0.0%) | 0.829 |
| CCTA, cardiac computed tomography angiography; PDL, peri-device leak; TEE, transesophageal echocardiography; DRT, device-related thrombosis. | | | | |
